# Supplementary material for: Evaluation of SARS-CoV-2 identification methods through surveillance of companion animals in SARS-CoV-2-positive homes in North Carolina, March to December 2020
Source: PeerJ. 2023 Oct 24;11:e16310. doi: 10.7717/peerj.16310 (PMC10607186; doi:10.7717/peerj.16310)
Supplement: Supplemental Information 1 [file peerj-11-16310-s001.docx]

**Additional PCR methodology**

SARS-CoV-2 RNA copies per milliliter were determined by a laboratory developed two-step real-time qPCR assay.  A QIAsymphony SP (Qiagen, Hilden, Germany) automated sample preparation platform along with a virus/pathogen DSP midi kit and the *complex800* protocol were used to extract viral RNA from 800 µL of oral or rectal swab samples.  A reverse primer specific to the envelope gene of SARS-CoV-2 (5’-ATA TTG CAG CAG TAC GCA CAC A-3’) was annealed to the extracted RNA and then reverse transcribed into cDNA using SuperScript^TM^ III Reverse Transcriptase (Thermo Fisher Scientific, Waltham, MA ) along with RNAse Out (Thermo Fisher Scientific, Waltham, MA ).  The resulting cDNA was treated with RNase H (Thermo Fisher Scientific, Waltham, MA ) and then added to a custom 4x TaqMan^TM^ Gene Expression Master Mix (Thermo Fisher Scientific, Waltham, MA ) containing primers and a fluorescently labeled hydrolysis probe specific for the envelope gene of SARS-CoV-2 (forward primer 5’-ACA GGT ACG TTA ATA GTT AAT AGC GT-3’, reverse primer 5’-ATA TTG CAG CAG TAC GCA CAC A-3’, probe 5’-6FAM/AC ACT AGC C/ZEN/A TCC TTA CTG CGC TTC G/IABkFQ-3’).  The qPCR was then carried out on a QuantStudio 3 Real-Time PCR System (Thermo Fisher Scientific, Waltham, MA).  SARS-CoV-2 RNA copies per reaction were interpolated using quantification cycle data and a serial dilution of a highly characterized custom RNA transcript containing the SARS-CoV-2 envelope gene sequence.  Mean RNA copies per milliliter were calculated by applying the assay dilution factor (DF=11.7).  The limit of quantification (LOQ) for this assay is approximately 62 RNA copies per milliliter of sample.  The primers and probes used in the described assay are from the Charité/Berlin World Health Organization (WHO) assay and are referred to as “E_Sarbeco.”
