# Supplementary material for: Evaluation of SARS-CoV-2 identification methods through surveillance of companion animals in SARS-CoV-2-positive homes in North Carolina, March to December 2020
Source: PeerJ. 2023 Oct 24;11:e16310. doi: 10.7717/peerj.16310 (PMC10607186; doi:10.7717/peerj.16310)
Supplement: Supplemental Information 2 [file peerj-11-16310-s002.docx]

| Canine Subject ID: ___________ | | | |
| --- | --- | --- | --- |
| Date of Consent: __ __/__ __ __ / __ __ __ __  dd /mm/ yyyy  Time of Consent: __ __: __ __  (hh:mm) (24 hour clock) | | - Electronic Consent | |
| Animal Breed |  | | |
| Animal Colorings/Markings |  | | |
| Sex | - Male | | - Female |
| Animal Neutered/Spayed | - Yes | | - No |
| Animal Age (approx.) |  | | |
| Pertinent Medical  History of Pet |  | | |
| Current Medications |  | | |
| Samples Obtained | - Oral swab - Fecal sample | | |
| Signature: _______________________________________________________________  Date:__________________ | | | |
