# Supplementary material for: Evaluation of SARS-CoV-2 identification methods through surveillance of companion animals in SARS-CoV-2-positive homes in North Carolina, March to December 2020
Source: PeerJ. 2023 Oct 24;11:e16310. doi: 10.7717/peerj.16310 (PMC10607186; doi:10.7717/peerj.16310)
Supplement: Supplemental Information 3 [file peerj-11-16310-s003.docx]

MESSI-HP Symptoms

| Subject ID: ______- ED -_________-____  Date of Collection: __ __ / __ ___ / ___ ___ ___ ___  dd /mmm/ yyyy | | | |
| --- | --- | --- | --- |
| **Time Point** (Please Circle One)  Historical symptom data (worst symptoms) - DATE: ________________________  Study Visit: Day 0 Day 7 Day 14 Day 21 Day 28 Month 2 Month 4 Month 6 Month 12  Sick Visit – DATE: _________________________ | | | |
| **Symptoms** | Symptom | Present | Not Present |
|  | Lethargy |  |  |
|  | Anorexia |  |  |
|  | Fever |  |  |
|  | Vomiting |  |  |
|  | Diarrhea |  |  |
|  | Sneezing |  |  |
|  | Coughing |  |  |
|  | Runny/watery eyes |  |  |
|  | Nasal discharge |  |  |
|  | Pale gums |  |  |
|  | Retching |  |  |
|  | Shortness of breath |  |  |
|  | Third eyelid raised |  |  |
