# Supplementary material for: Evaluation of SARS-CoV-2 identification methods through surveillance of companion animals in SARS-CoV-2-positive homes in North Carolina, March to December 2020
Source: PeerJ. 2023 Oct 24;11:e16310. doi: 10.7717/peerj.16310 (PMC10607186; doi:10.7717/peerj.16310)
Supplement: Supplemental Information 4 [file peerj-11-16310-s004.docx]

**Animal Contact**

Do you currently have any of the following animals as a pet? (Please select all that apply)

Dog Cat Ferret Other (please specify)

If yes, how long have you owned each pet?

If yes, please indicate which of the following applies for each pet:

1. Exclusively indoors (does not spend any time outdoors)
2. Outdoors with supervision only (is never outdoors without supervision of owner)
3. Confined outdoors without supervision (spends time outdoors without direct supervision, but is confined to owners property via fencing, tethers, or other means)
4. Roams outdoors (allowed to roam freely outdoors)

How would you classify the area where you live?

Urban Suburban Rural

For each pet, do they…

Share your bed?

Share any furniture with you?

Lick your hands?

Lick your face?

Lick other body parts? (please specify body part)

Eat from your plates/dishes?

Hunt or bring prey for you? (please specify prey)

Have you been bitten by any of your pets in the past month?

If yes, which pet and when did the bite occur?

Have you been scratched by any of your pets in the past month?

If yes, which pet and when did the bite occur?

Have any of your pets been ill in the past month?

If yes, which pet and specify symptoms

Aside from your own pets, have you had contact with any of the following animals through hobbies, activities, work, or volunteer work in the past month?

Dog Cat Ferret Other (please specify)

For any of these animals, have you been bitten, scratched, or licked?

If yes, please specify:

Have you had contact with any of the following animals through your job in the past month?

Dog Cat Ferret Other (please specify)

For any of these animals, have you been bitten, scratched, or licked?

If yes, please specify:
