# Supplementary material for: Evaluation of SARS-CoV-2 identification methods through surveillance of companion animals in SARS-CoV-2-positive homes in North Carolina, March to December 2020
Source: PeerJ. 2023 Oct 24;11:e16310. doi: 10.7717/peerj.16310 (PMC10607186; doi:10.7717/peerj.16310)
Supplement: Supplemental Information 5 [file peerj-11-16310-s005.docx]

| **Species** | **Day sampled** | **Symptoms? (Y/N)** | **Sample type** | **Test performed** | **Result** |
| --- | --- | --- | --- | --- | --- |
| **Dog** | 7 | N | Oral swab | PCR | Negative |
|  | 21 | N | Oral swab | PCR | Negative |
|  | 21 | N | Rectal swab | PCR | Negative |
|  | 28 | Y, lethargy, itchy/watery eyes | Oral swab | PCR | Negative |
|  | 28 |  | Rectal swab | PCR | Negative |
|  | 120 | N | Serum | ELISA | Positive |
| **Dog** | 0 | Y, coughing | Oral swab | PCR | Negative |
|  | 21 | Y, lethargy | Oral swab | PCR | Negative |
|  | 21 | Y, lethargy | Rectal swab | PCR | Negative |
|  | 120 | N | Serum | ELISA | Positive |
| **Dog** | 0 | Y, runny nose | n/a | n/a | n/a |
|  | 3 | N | Oral swab | PCR | Negative |
|  | 3 | N | Rectal swab | PCR | Negative |
|  | 28 | N | Oral swab | PCR | Negative |
|  | 28 | N | Rectal swab | PCR | Negative |
|  | 28 | N | Serum | ELISA | Positive |

Supplementary materials 5: Overview of testing timeline and symptoms present in animals with positive IgG serology for SARS-CoV-2 and the presence of owner-reported symptoms on at least one timepoint.
